# *Irg1*/itaconate metabolic pathway is a crucial determinant of dendritic cells immune-priming function and contributes to resolute allergen-induced airway inflammation

**DOI:** 10.1038/s41385-021-00462-y

**Published:** 2021-10-20

**Authors:** Anil Kumar Jaiswal, Jyoti Yadav, Sangeet Makhija, Suman Mazumder, Amit Kumar Mitra, Amol Suryawanshi, Maninder Sandey, Amarjit Mishra

**Affiliations:** 1 From the Laboratory of Lung Inflammation, Auburn University, Auburn, AL USA; 2 Department of Pathobiology, College of Veterinary Medicine, Auburn University, Auburn, AL USA; 3 Department of Drug Discovery and Development, Auburn University, Auburn, AL USA; 4 Center for Pharmacogenomics and Single-Cell Omics, Harrison School of Pharmacy, Auburn University, Auburn, AL USA

## Abstract

Itaconate is produced from the mitochondrial TCA cycle enzyme aconitase decarboxylase (encoded by immune responsive gene1; *Irg1*) that exerts immunomodulatory function in myeloid cells. However, the role of the *Irg1*/itaconate pathway in dendritic cells (DC)-mediated airway inflammation and adaptive immunity to inhaled allergens, which are the primary antigen-presenting cells in allergic asthma, remains largely unknown. House dust mite (HDM)-challenged *Irg1*^*−/−*^ mice displayed increases in eosinophilic airway inflammation, mucous cell metaplasia, and Th2 cytokine production with a mechanism involving impaired mite antigen presentations by DC. Adoptive transfer of HDM-pulsed DC from *Irg1*-deficient mice into naïve WT mice induced a similar phenotype of elevated type 2 airway inflammation and allergic sensitization. Untargeted metabolite analysis of HDM-pulsed DC revealed itaconate as one of the most abundant polar metabolites that potentially suppress mitochondrial oxidative damage. Furthermore, the immunomodulatory effect of itaconate was translated in vivo, where intranasal administration of 4-octyl itaconate 4-OI following antigen priming attenuated the manifestations of HDM-induced airway disease and Th2 immune response. Taken together, these data demonstrated for the first time a direct regulatory role of the *Irg1*/itaconate pathway in DC for the development of type 2 airway inflammation and suggest a possible therapeutic target in modulating allergic asthma.

## Introduction

Asthma is a heterogeneous disease of upper airways with an increasing prevalence rate (>25 million in the United States), resulting in high morbidity and cost to the health care system^1,2^. Multiple pathobiological subtypes of asthma patients manifest the heterogeneity of airway inflammation, complicating the response to therapy and impacting health outcomes^3–5^. Although inhaled corticosteroids (ICS) have been the foundation of conventional asthma therapy and management, ~10% of asthmatic patients develop severe steroid-refractory asthma with mixed granulocytic inflammation comprising both neutrophils and eosinophils^6–8^.

The mitochondria-selective metabolite itaconic acid is produced from decarboxylation of TCA cycle intermediate cis-aconitate and is encoded by immune responsive gene1 (*Irg1*, also known as *ACOD1*)^9^. Besides its canonical antibacterial role through isocitrate lyase inhibition^10^, the *Irg1*/itaconate pathway also regulates inflammation and infections^11–16^. For example, the receptor-interacting protein kinase (RIPK)-dependent antiviral responses are coupled to *Irg1* as deletion of *Irg1* shows increased neuronal viral load and mortality rate in zika virus (ZIKV) and West Nile Virus (WNV) infections^17,18^. Upregulation of the *Irg1*/itaconate pathway is linked with several inflammatory diseases, including sepsis^19,20^, psoriasis^21^, glioma^22,23^ peritoneal tumors^24^, abdominal aortic aneurysm^25^, hepatic ischemia-reperfusion injury^26^, and pulmonary fibrotic conditions^27^. Moreover, myeloid cell-specific *Irg1* ablation increases the susceptibility to *Mycobacterium tuberculosis* infection and lung immunopathology^28^.

The antigen-presenting dendritic cells (DC) instigates and regulates different types of effector T cells (Teff) responses based on the context they sense the antigen, which is necessary and sufficient for inducing Th2- and Th1/Th17 adaptive immunity to inhaled allergens. DC activation links to profound metabolic changes that impact immune-priming and immune-polarizing effector function(s)^29,30^. In addition to diverse molecular mechanisms, efficient antigen processing requires protection of potential T-cell epitopes from degradation and is markedly influenced by intracellular reactive oxygen species (ROS) productions^31,32^. Itaconate is a strong electrophile and able to alkylate cysteine residues in various target proteins. A potent antioxidant and immunomodulatory function of itaconate have been suggested to several inflammatory conditions due to its ability to induce transcription factor nuclear factor erythroid 2-related factor 2 (Nrf2) expression^33^. Consistent with this notion, the cell-permeable surrogates dimethyl ester (DI) or 4-octyl ester (4-OI) of itaconate have been demonstrated to suppress ROS-dependent oxidative damage via induction of antioxidant genes including NQO1 and HO-1 as well as succinate dehydrogenase (SDH) blockade^33,34^. Most interestingly, derivatives of itaconate abrogate LPS-induced endotoxemia and psoriasis partly by activating transcription factor 3 (ATF3)-dependent induction of the negative regulator IkBζ^21,35^. 4-OI-induced elevated Nrf2 expression is associated with antiviral response suppressing the downstream adaptor protein stimulator of interferon genes (STING) and IFNs^36–38^. Still, a better understanding of the *Irg1*/itaconate pathway that regulates DC effector function(s) and immune response development in airway type 2 inflammation remains largely unexplored.

Akin to itaconate, previously, we have shown that dimethyl fumarate (DMF; α, β-unsaturated carboxylic acid ester of TCA cycle intermediate fumarate) treatment markedly attenuates house dust mite (HDM)-mediated airway inflammation and development of Th2 immune response^39^. In particular, we demonstrate that DMF interferes with airway DC migration to draining mediastinal lymph nodes (MedLN), thereby modulating eosinophilic airway inflammation and allergic asthma. Since the *Irg1*/itaconate pathway is essential for innate immune response and inflammation, we hypothesized whether it impacts the adaptive immune responses to aeroallergen HDM. Moreover, we investigated whether exogenous administration of 4-OI can attenuate airway type 2 inflammation in asthma. To examine the role of the *Irg1*/itaconate pathway in asthma, we utilize repeated HDM challenge with low dose STING agonist cyclic-di-GMP (c-di-GMP) administration, which is a mucosal adjuvant and acts as an innate immune sensor. Our results indicate that HDM and STING stimulation progressively activates *Irg1* in DC via a mitochondrial superoxide-dependent pathway and impairs effective antigen presentation, HDM-induced airway type 2 inflammatory response. Our result provides a critical link between the *Irg1*/itaconate pathway and induction of Th2-immunity in allergic asthma. The findings also implicate novel insight into the molecular mechanisms of airway type 2 inflammation and allergic asthma that might be fundamental for developing metabolism-based therapeutic options.

## Results

### *Irg1* expression is elevated in HDM-induced airway inflammation

**Fig. 1:**
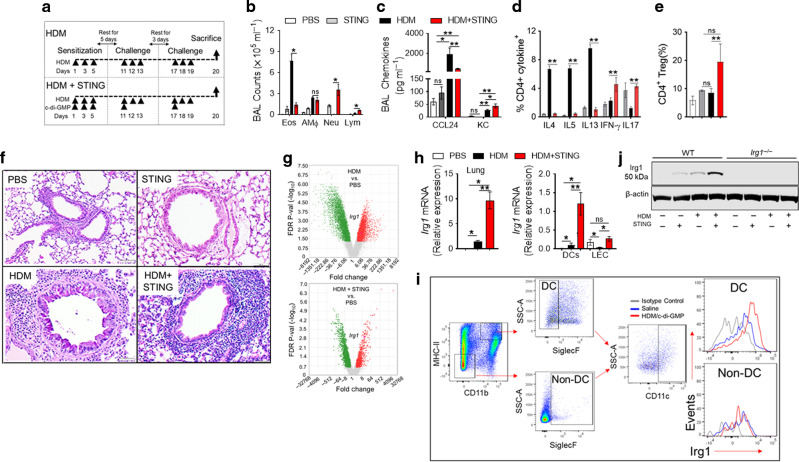
*Irg1* is induced in DC associated with asthma. **a** Wild-type mice were sensitized and challenged by administration of PBS, HDM (50 μg), STING agonist c-di-GMP (5 μg), and HDM + STING, as shown. Endpoint analysis was performed 24 h after the last administration of HDM. **b** The number of BAL inflammatory cell types (eosinophils (Eos), alveolar macrophages (AMΦ), neutrophils (Neu), and lymphocytes (Lym)) from PBS−, STING−, HDM− and HDM + STING-challenged were compared. (*n* = 6–10 mice represent one of the three independent experiments; *P < 0.01, HDM versus HDM + STING, one-way ANOVA with Sidak’s multiple comparison test). Bar charts shows (**c**) BAL levels of CCL24 and KC chemokines, frequency of (**d**) CD4^+^ cytokines^+^ T cells and (**e**) CD4^+^ CD25^+^ Foxp3^+^ Treg cells in medLN (*n* = 8–10 mice, *P < 0.05, *P*BS versus HDM or HDM + STING, **P < 0.01, HDM versus HDM + STING, one-way ANOVA with Sidak’s multiple comparison test). **f** Representative histologic lung sections stained with PAS. *Scale bars*, 100 μm for ×100 images. **g** Volcano plot representing FDR-p values of significant (FDR < 0.05) DE genes. *A pink circle marks irg1*. **h** qPCR showing relative expression of *Irg1* in lungs and in isolated CD11c^+^ DCs, and Epcam^+^ lung epithelial cells (LEC^)^ normalized to ^β^-actin RNA. **i** Representative histogram showing *Irg1* expressions on live lung cells gated on DC (SiglecF^−^/ CD11c^+^/ CD11b^+^/ MHCII^hi^) and non-DC (SiglecF^+^/ CD11c^+^/ CD11b^−^/ MHCII^−^ population. (*n* = 4–6 mice, represent one of the three independent sets of experiments; *P < 0.05, PBS versus HDM or HDM + STING, **P < 0.01, HDM versus HDM + STING, one-way ANOVA with Sidak’s multiple comparison test) **j** Western blot showing Irg1 protein induction in BMDC isolated from WT and *Irg1*^*−/−*^ mice and pulsed with HDM (100 μg/ ml) and STING agonist, c-di-GMP (5 µM) and stimulated for 16 h. Results show the mean ± SD and represent two or more independent experiments.

Upper respiratory infections commonly trigger asthma exacerbations increasing type I IFN signaling through STING pathways^40,41^. Stimulation of the lung with the aeroallergen HDM and STING agonist c-di-GMP induces mixed granulocytic airway inflammation accompanied by Th2-low and Th1/Th17-high immune responses^42,43^. To study the role of the *Irg1*/itaconate pathway in airway inflammation, we first employed two murine models of experimental asthma that mirror the endotypes manifested in asthma patients^42,44–46^ (Fig. 1a). Twenty-four hours after the conclusion of the last allergen challenge, airway inflammation and immune responses were evaluated employing flow cytometry. The results showed that HDM-challenged mice had increases in eosinophilic airway inflammation than HDM + STING-challenged mice, which accumulated both neutrophils and eosinophils as mixed granulocytic infiltrate (Fig. 1b). C-C chemokine CCL24, which recruits eosinophils, or C-X-C chemokine KC, which recruits neutrophils, was significantly increased in BAL with HDM-challenge alone or STING stimulation compared with naïve PBS or STING alone control mice, respectively (Fig. 1c). HDM stimulation alone showed significant infiltrations of CD4^+^ effector T cells expressing high Th2 signature cytokines and low Th1/Th17, whereas reversed in the presence of combined HDM and STING stimulation (Fig. 1d). The frequency of Foxp3^+^ regulatory T cells (Tregs) in MedLN was markedly increased in both conditions compared to PBS or STING-treated naïve mice (Fig. 1e). Moreover, lung histology showed that the mice subjected to both HDM and STING stimulation harbored more intense peribronchial inflammatory cell infiltrates compared with those sensitized and challenged with HDM alone (Fig. 1f). Next, we employed a microarray analysis of the lung transcriptome to identify shared molecular signatures in these Th2-high and Th2-low experimental asthma models. Gene expression analysis of allergic lungs showed a total of 12,673 genes (6549 upregulated and 6124 downregulated) were differentially regulated in PBS vs. HDM compared to 5000 genes (1739 upregulated and 3261 downregulated) in PBS vs. HDM + STING (Supplementary Fig. 1a, b). Among these, 4078 genes were shared between the two comparisons (Supplementary Fig. 1c). We focused on immune responsive gene 1 (*Irg1*) amongst the upregulated genes in HDM alone, or HDM and STING pathway stimulated lungs compared with PBS control lungs (Fig. 1g). *Irg1* encodes for the mitochondrial enzyme aconitase decarboxylase that drives the decarboxylation of TCA cycle intermediate cis-aconitate for itaconic acid production^9^. Lungs from both groups showed significant induction of *Irg1* as compared with naïve lung, which was further confirmed by qPCR analysis (Fig. 1h). Moreover, Ingenuity Pathway Analysis (IPA) based on the most significant DE genes revealed additional molecular pathways differentially regulated between the subgroups^47^. For example, IPA- predicted inhibition of IL10RA as the top upstream molecule based on the gene expression profiling (GEP) signature includes *Irg1* (Supplementary Fig. 1d). Because DC induces and regulates different types of effector T cells response and develops Th2- and Th1/Th17 adaptive immunity to inhaled allergens, we hypothesized that *Irg1* expression and itaconate production by DC might regulate T cell-mediated immune response in HDM-induced inflammatory response. We found that HDM + STING stimulation markedly induced *Irg1* expression and is restricted to lung CD11c^+^ SiglecF^−^ CD11b^+^ MHCII ^hi^ DC population compared with CD11c^+^ SiglecF^+^ CD11b^−^ MHCII ^lo^ non-DC subset of cells (Fig. 1i). Furthermore, Western blots of HDM-pulsed bone-marrow-derived DC (BMDC) proteins demonstrated that HDM and STING stimulation markedly induces *Irg1* expression, which is absent in *Irg1*^*−/−*^ BMDC (Fig. 1j). Overall, these results collectively showed that HDM sensitization and STING pathway stimulation progressively induces the expression of *Irg1* in allergic lungs and DC.

### Deletion of *Irg1* augments HDM-induced eosinophilic airway inflammatory response

**Fig. 2:**
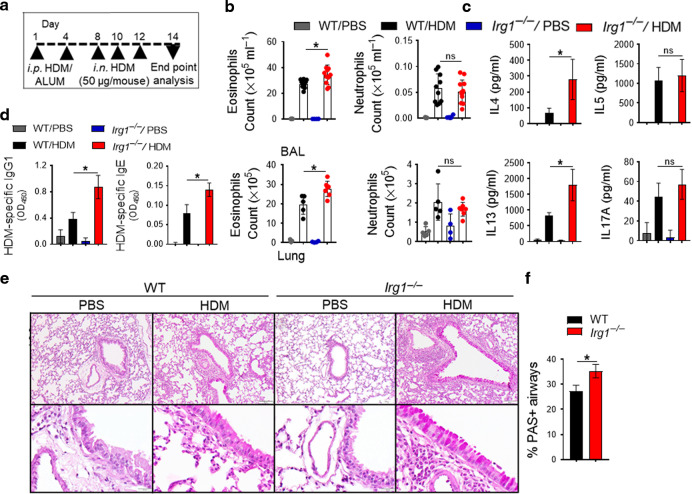
Th2 cytokine production and allergic sensitization are increased in HDM-challenged *Irg1*^*−/−*^ mice. HDM was administered to WT (black) or *Irg1*^*−/−*^ (red) mice. **a** The diagram shows the HDM administration (*i.p*., intraperitoneal; *i.n*., intranasal) and analysis schedule. **b** Scatter plot with bar show the numbers of CD11b^+^ Siglec-F^+^ eosinophils and CD11b^+^ Ly-6G^+^ neutrophils in BAL (top) and lung (bottom). **c** Bar charts show the cytokine secretion by ex vivo cultures of MedLN cells that had been re-stimulated with HDM (100 μg ml^−1^), **d** Serum HDM-specific IgE and IgG1 levels. **e** Images show histopathological sections of HDM-challenged lung from WT or *Irg1*^*−/−*^ stained with PAS. *Scale bars*, 100 μm for ×100 and 50 μm for ×400 images. **f** Enumerated PAS^+^ airways from histopathological lung sections (*n* = 5–7 mice, one of 3 representative experiments is shown; *P < 0.05, WT versus *Irg1*^*−/−*^, unpaired *t-*test, two independent experiments).

Whether deletion of *Irg1* similarly modifies the induction of HDM sensitization and Th2 immune response, experiments were next conducted with multiple nasal HDM challenges to wild-type (WT) and *Irg1*^*−/−*^ mice (Fig. 2a). Our results demonstrate a significant increase in total BAL and lung inflammatory cells in HDM-challenged *Irg1*^*−/−*^ mice, which reflected a selective increase in eosinophils. However, the numbers of neutrophils were not altered (Fig. 2b). Restimulation of MedLN cells with HDM isolated from inflamed *Irg1*^*−/−*^ mice showed a significant increase in IL4 and IL13 Th2 cytokines production compared with HDM-challenged WT. In contrast, the increase in IL5 production was not statistically significant (Fig. 2c). IL17A production by MedLN cells following ex vivo HDM restimulation is not substantial. Because CD11b^+^ conventional DC in the airways is known for the majority of the antigen presentation and Th2 responses^48–50^, we further characterized these DC subsets in the draining MedLN. We found that CCR7, CD80 (co-stimulatory molecule), Dectin2 (CLRs), and CD205 (antigen uptake) surface expressions were significantly increased in HDM-challenged *Irg1*^*−/−*^ mice compared with WT. In contrast, there were no differences in CD86, Dectin1, and TLR4 mean fluorescence intensity of CD11b^+^ migratory DCs in MedLN (Supplementary Fig. 2a, b). HDM-specific IgE and IgG1 levels in serum were significantly elevated in *Irg1*^*−/−*^ mice compared to HDM-challenged WT mice, thereby demonstrating increased susceptibility of *Irg1*^−/−^ mice to induce allergic sensitization (Fig. 2d). Moreover, we found an increase in peribronchial inflammatory cell infiltrates in HDM-challenged *Irg1*^*−/−*^ mice lungs and in PAS^+^ airways as compared with WT mice controls (Fig. 2e, f). Collectively, these results demonstrate that *Irg1*^*−/−*^ mice have a phenotype of increased eosinophilic airway inflammation, mucous cell metaplasia, and Th2-mediated immune responses in HDM-induced asthma.

### HDM-pulsed BMDC adoptive transfer from *Irg1*^*−/−*^ mice accelerates airway inflammation in WT recipient mice

**Fig. 3:**
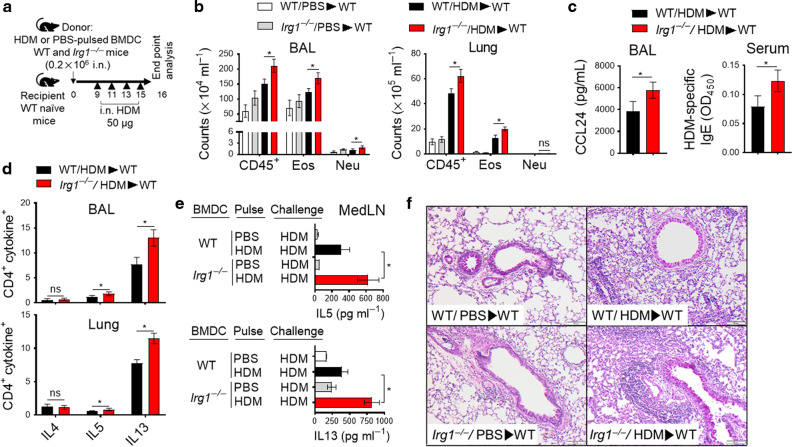
Adoptive transfer of BMDC from *Irg1*^*−/−*^ mice promotes HDM-mediated type 2 airway inflammation in WT mice. **a** BMDC from WT or *Irg1*^*−/−*^ mice were HDM-pulsed (100 μg/ ml) or sham-pulsed with PBS for 16 h and were adoptively transferred to naïve WT mice (0.2 × 10^6^ cells in 40 μl sterile saline; *i.n*. administration). Recipient WT mice received HDM challenges (50 μg; i.n) on days 9, 11, 13, and 15 before harvest on day 16. **b** The number of total CD45+ inflammatory cells, eosinophils, and neutrophils in recipient WT BAL and lung; **c** BAL CCL24 (left) and serum levels of HDM-specific IgE (right) and **d** quantification of Th2 immune response, as assessed based on the percentage of CD4+cytokinev+ cells in BAL and lung. **e** IL5 and IL13 cytokine secretion in culture supernatants of MedLN cells were re-stimulated with or without HDM (100 μg/ml). **f** Representative histopathological sections of PAS-stained WT recipient lungs. *Scale bars*, 100 μm for ×100. Data are expressed as mean ± SEM, and one of the three adoptive transfer experiments is shown; (*n* = 4–5 mice per group, *P < 0.05, WT/HDM/WT versus *Irg1*^*−/−*^/ HDM/ WT, unpaired *t-*test).

Next, adoptive transfer experiments were performed to examine the role of DC explicitly contributing to the accelerated airway inflammatory responses in *Irg1*^*−/−*^ mice. As shown in Fig. 3a, BMDC from WT and *Irg1*^*−/−*^ mice were pulsed with HDM (100 µg ml^−1^) or sham-pulsed with PBS. Naïve WT mice received BMDC from WT and *Irg1*^*−/−*^ mice and were challenged with multiple HDM to instigate type 2 airway inflammation. The total number of BAL and lung CD45^+^ cells, predominantly composed of eosinophils, was significantly elevated in the WT recipient mice receiving *Irg1*^*−/−*^ BMDC compared with WT BMDC (Fig. 3b). BAL CCL24 (C-C chemokine that interacts with CCR3 to recruit eosinophils) and HDM-specific IgE levels in serum were significantly elevated in WT recipients of *Irg1*^*−/−*^ BMDC compared with WT recipients of WT BMDC (Fig. 3c). In addition, the proportion of CD4^+^ Th2 cytokine+ (IL5^+^ and IL13^+^) cells in BAL and lungs from WT recipient mice that received *Irg1*^*−/−*^ BMDC were markedly increased; however, CD4^+^ IL4^+^ cells remain unchanged as compared to WT recipient mice that received WT BMDC (Fig. 3d). The supernatants of HDM-restimulated cultures of MedLN cells from recipient mice that received *Irg1*^*−/−*^ donor BMDC showed higher IL5 and IL13 cytokine production than those of the WT donor BMDC (Fig. 3e). Similarly, lung histology revealed increases in peribronchial and perivascular inflammatory cell infiltrates in recipient WT mice receiving *Irg1*^*−/−*^ donor BMDC compared with WT recipients of WT donor BMDC (Fig. 3f). These results collectively restate the enhanced airway inflammation phenotype observed in HDM-challenged *Irg1*^*−/−*^ mice. They are consistent with the conclusion that the *Irg1/*itaconate pathway plays a pivotal role in DC effector function, contributing to HDM-induced type 2 airway inflammation and allergic asthma.

### Deletion of *Irg1* impacts mitochondrial function in DC

**Fig. 4:**
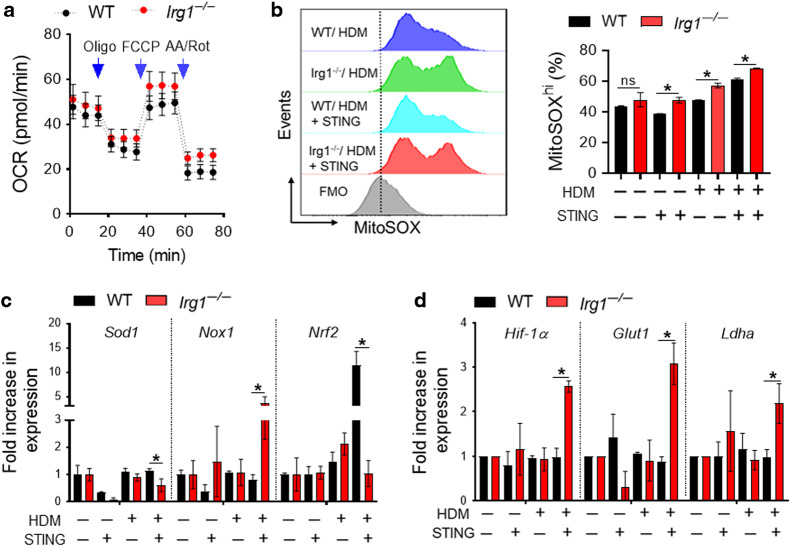
Increase mitochondrial oxidative stress in *Irg1*-deficient DC. **a** Extracellular flux was analyzed on HDM-pulsed BMDC isolated from WT (black) and *Irg1*^*−/−*^ (red) mice. A metabolic stress test was performed, and kinetic oxygen consumption rate (OCR) was graphed in response to sequential treatments of oligomycin (Oligo, 1 mM), carbonyl cyanide 4-(trifluoromethoxy) phenylhydrazone (FCCP, 2 mM), and antimycin A/rotenone (0.5 mM) after basal measurements. Each circle represents the mean ± SE (*n* = 6). **b** Histogram overlays (left) and bar graph frequencies of MitoSOX^hi^ CD11c^+^ CD11b^+^ BMDC (right) pulsed with HDM or HDM + STING. qPCR for mRNA expression of (**c**) antioxidant and (**d**) metabolic genes. (*n* = 4–6 per group, *P < 0.05, WT versus *Irg1*^*−/−*^, unpaired *t-*test). Data represent the mean ± SD of at least two independent experiments.

Upon allergen stimulation, matured DC exhibit metabolic reprogramming that impacts immune-priming functions in asthma development^30,51–53^. Because *Irg1*/itaconate pathway is coupled to mitochondrial oxidative phosphorylation, we next examined whether *Irg1* deficiency in HDM-pulsed BMDC would alter oxygen consumption rate (OCR) and mitochondrial respiratory activity. In the presence of HDM, WT BMDC showed typical OCR changes in response to sequential inhibition of mitochondrial ATP synthase by oligomycin, uncoupling of OXPHOS from ATP synthesis with FCCP, ETC inhibition by antimycin/rotenone, whereas *Irg1*^*−/−*^ BMDC had increased mitochondrial function as measured by XFp seahorse analyzer (Fig. 4a). Next, we considered whether HDM and STING stimulation could induce oxidative stress and superoxide generation as a mechanism by which *Irg1* is coupled with mitochondrial oxidative function. As shown in Fig. 4b*, Irg1*^*−/−*^ BMDC mediate a significant increase in mitochondrial superoxide production after HDM pulse with or without STING stimulation. In addition, there was a substantial reduction in superoxide dismutase 1 (SOD1) expression in *Irg1*^−/−^ BMDC compared with WT, which are known scavengers of superoxide radicals (Fig. 4c). Additionally, the superoxide-generating oxidase *Nox1* expression by *Irg1*^*−/−*^ BMDC was significantly increased, whereas the expression of master antioxidant transcription factor *Nrf2* has decreased with HDM and STING stimulation. *Irg1*-deficient DC showed augmented expression of metabolic genes including *Hif-1α*, *Glut1*, and *Ldha* compared with WT (Fig. 4d). Collectively, these data suggest that elevated oxidative stress and mitochondrial function in HDM-pulsed *Irg1*^*−/−*^ DC might partly drive the development of enhanced airway type2 inflammation.

### *Irg1*-deficient DC have enhanced antigen priming capability

**Fig. 5:**
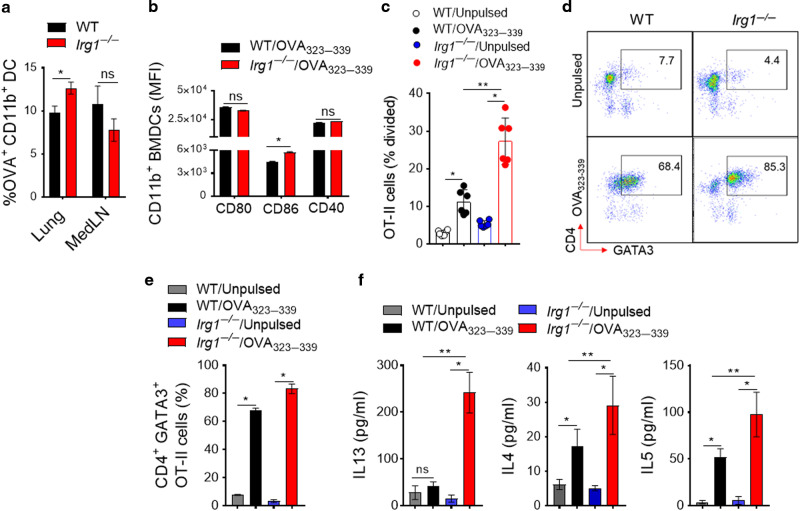
*Irg1*-deficient DC has augmented antigen priming and presentation capabilities. **a** The proportion of OVA-AF647+CD11b+DC (gated on SiglecF^−^ CD11c^+^ MHCII^hi^ population) in the lung and MedLN of WT and *Irg1*^*−/−*^ mice 72 h after the allergen administration. Results represent the mean ± S.E, pooled from two independent experiments (*n* = 10 mice per group, *P < 0.05 unpaired *t-*test). **b–f** CD11c^+^ CD11b^+^ BMDC from WT and *Irg1*^*−/−*^ mice were pulsed with the OVA_323-339_ peptide and incubated at 1: 5 ratio with CFSE-labeled splenic OT-II cells for 4 days. **b** Surface expression (Mean fluorescence intensities, MFI) of activation markers in BMDC stimulated with OVA_323-339_ peptide. **c** OVA-specific proliferation is presented as a percentage divided OT-II cells. Each circle represents individual mice. **d** Pseudocolor plots show representative flow cytometric data, and **e** bar graphs show frequencies of CD4^+^ GATA3^+^ OT^-^II cells in coculture. **f** Th2 cytokines released by cocultures of OVA_323-339_ pulsed BMDC and CFSE-labeled OT-II cells (*n* = 6 per group and represent one of the three repeats;). * Data represent the mean ± SD, *P* < 0.01, and **P < 0.05 one-way ANOVA with Sidak’s multiple comparison test.

We next assessed whether the antigen priming capabilities of *Irg1*-deficient DC could interfere with the uptake and presentation of allergen as a possible mechanism of increased Th2/eosinophilic polarized HDM allergic responses in *Irg1*^*−/−*^ mice. AF647-labeled OVA was administered intranasally to the WT and *Irg1*^*−/−*^ lung, and the uptake/ migration of OVA-loaded CD11b^+^ DC to MedLN were enumerated by flow cytometry after 72 h. As shown in Fig. 5a, frequencies of OVA bearing Alexa 647 + CD11b^+^ DC in the lung showed a significant increase in *Irg1*^*−/−*^ mice compared with WT. In contrast, no significant differences were found between the genotypes in the frequency of CD11b+OVA-AF647+DC that migrated to the draining MedLN after OVA antigen uptake by the lung, suggesting no migratory defects of *Irg1*^*−/−*^ DCs as compared to WT. Furthermore, stimulation with OVA_323-339_ peptide induces surface expression of CD86 in BMDC isolated from *Irg1*^*−/−*^ compared to WT, whereas changes in CD40 and CD80 surface expression were modest. Coculture of CD4^+^ T-cells from OT-II transgenic mice that discerns the OVA_323-339_ peptide displayed a marked increase in T-cell proliferation (Fig. 5c) and GATA3 (Th2-specific transcription factor) expression (Fig. 5d, e) in the presence of *Irg1*^*−/−*^ BMDC compared with WT BMDC after OVA_323-339_ peptide stimulation. Similarly, BMDC from *Irg1*^*−/−*^ mice showed augmented ability to induce Th2 cytokines, IL13, and IL4 productions compared with WT BMDC following OVA_323-339_ peptide stimulation when cocultured with splenic T-cells obtained from OT-II mice (Fig. 5f). Collectively, these in vivo and ex vivo results show that *Irg1*-deficient DC has an enhanced allergen-uptake and antigen-presenting capability to CD4^+^ T-cells and thereby accelerates Th2 effector functions.

### 4-OI restores mitochondrial redox in DC

**Fig. 6:**
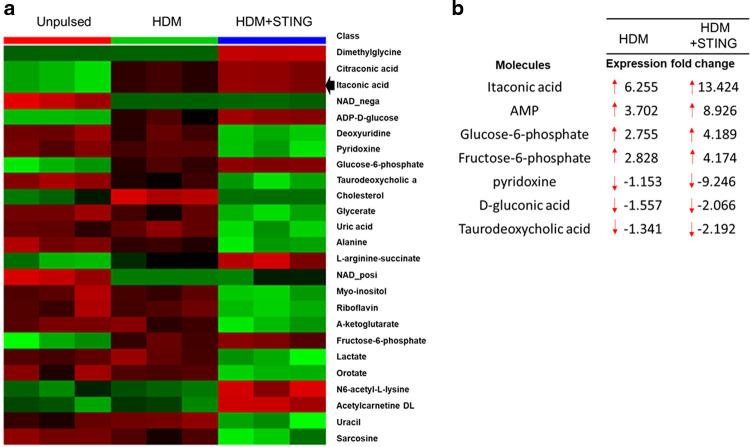
Induction of itaconate production in HDM-pulsed BMDC. **a** Heatmap of selected top 25 metabolites ranked by *t*-test (*P* < 0.05) and **b** IPA of the unbiased metabolomic analysis of extracted polar metabolites from HDM(100 µg ml^−1^)-pulsed BMDC treated with or without STING agonist c-di-GMP (10 µM) for 16 h. Quantitation of polar metabolites was performed using positive/negative ion-switching LC-MS/MS with a 5500 QTRAP hybrid triple quadrupole mass spectrometer. Each group contained at least 3 replicate samples. The data set was analyzed using IPA software to obtain a list of the top-selected upregulated and downregulated metabolites in HDM-pulsed BMDC treated with and without STING compared with the unpulsed control.

Since HDM significantly instigated *Irg1 expression* in DC, we next investigated whether changes in metabolite levels are associated with HDM and STING stimulation. Employing untargeted primary metabolites profiling in BMDC, we identified several metabolites that were significantly upregulated by HDM alone or with STING stimulation compared with unpulsed BMDC (Fig. 6a). Surprisingly, using IPA analysis of the metabolomics data, we found that BMDC pulsed with HDM and STING (>13-fold) or HDM alone (>6-fold) accumulates itaconic acid as one the most highly abundant metabolite (Fig. 6b).

**Fig. 7:**
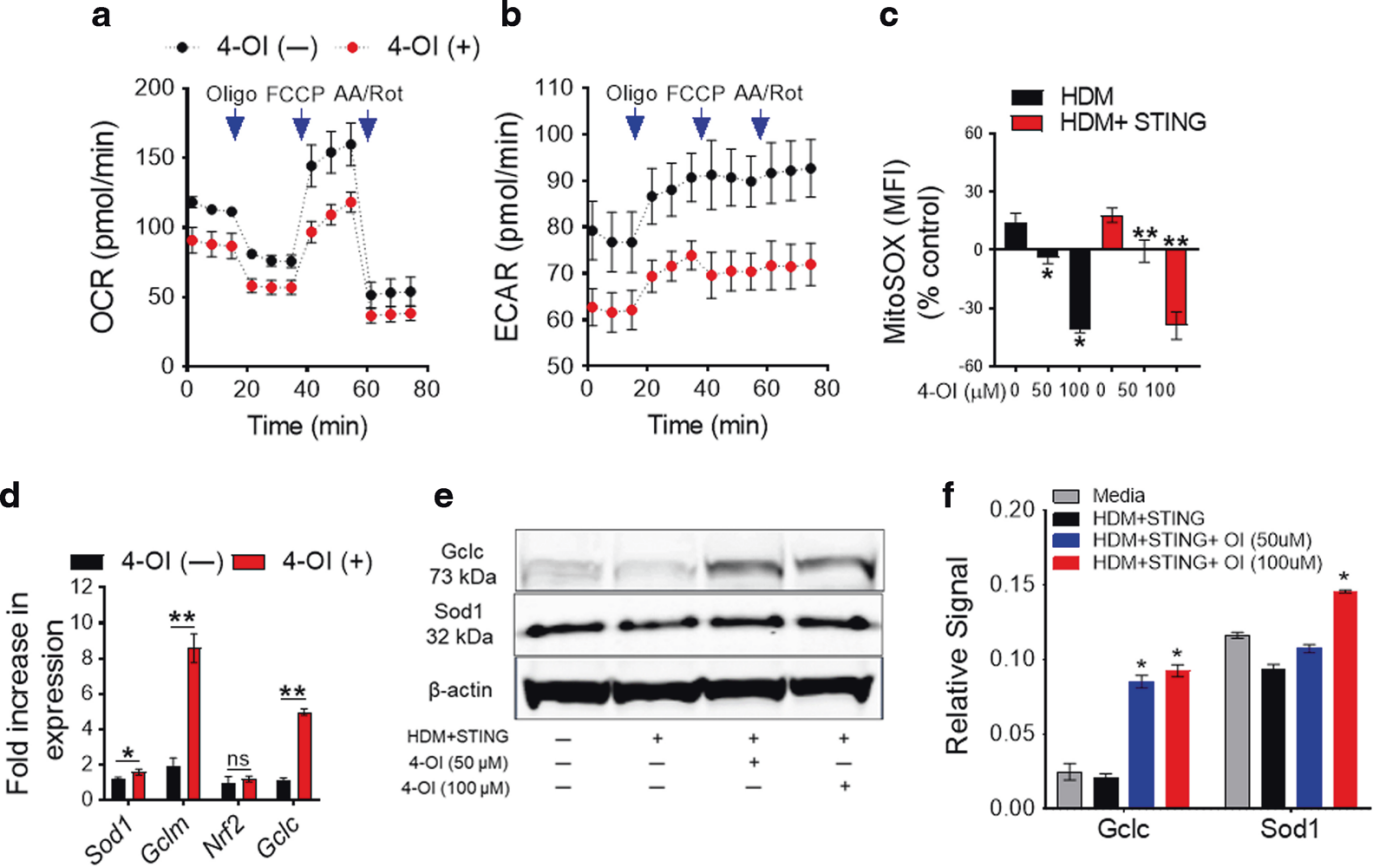
4-OI attenuates mitochondrial oxidative metabolism and superoxide generation. Metabolic flux (XFp) of a mitochondrial stress test representing (**a**) OCR and (**b**) ECAR were graphed over time. Arrowhead indicates drug injection point. HDM and STING stimulated BMDC were incubated with 4-OI (50 µM) for 18 h before the XFp assay. All plots represent one of three experiments. **c** A bar graph shows the percent change in mean fluorescence intensity of MitoSOX^+^ CD11b^+^ BMDC treated with different doses of 4-OI compared to unstimulated control. **d** qPCR of antioxidant genes and **e** representative western blot of HDM and STING-stimulated BMDC with or without 4-OI. Data represent the mean ± S.E from one experiment out of three independent repeats (*n* = 6, *P < 0.05 and **P < 0.01 one-way ANOVA with Sidak’s multiple comparison test).

Next, experiments were conducted to assess whether HDM and STING pulsed-BMDC pretreated with itaconate derivative 4-OI have restored mitochondrial oxidative damage. 4-OI treatment significantly reduces oxygen consumption rates (OCR) and extracellular acidification rates (ECAR) as compared with untreated control in BMDC stimulated with HDM and STING (Fig. 7a, b). Furthermore, 4-OI markedly attenuates mitochondrial superoxide generation by HDM- and STING stimulation in a dose-dependent manner as measured by MitoSOX oxidation using flow cytometry (Fig. 7c). Mechanistically, itaconate derivatives activate the transcription factor Nrf2 via alkylation of the KEAP protein residues, leading to the upregulation of downstream Nrf2-dependent antioxidant genes such as *SOD1*, *Gclc*, and *Gclm*^33,54^. Consistent with this, 4-OI treatment in HDM and STING-pulsed BMDC induces *Sod1, Gclm*, and *Gclc* expressions at mRNA and protein levels, suggesting that 4-OI restores mitochondrial redox promoting an antioxidant program (Fig. 7d–f). Overall, these results agree that the *Irg1*/itaconate pathway is crucial for mitochondrial redox change and could be manipulated by exogenous 4-OI treatment.

### Exogenous itaconate administration attenuates the cardinal manifestations of HDM-induced airway inflammation

**Fig. 8:**
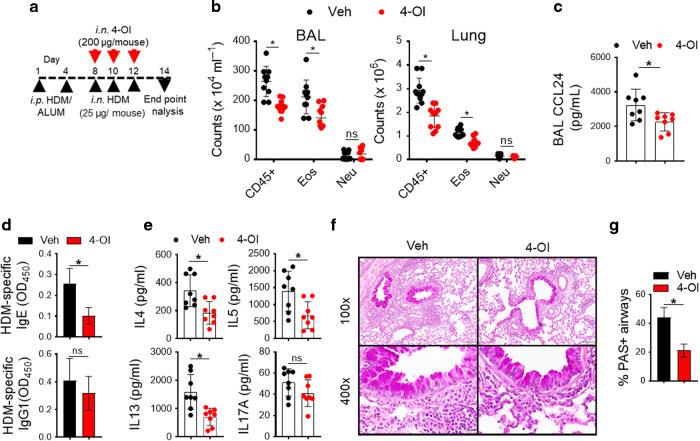
Intranasal administration of the exogenous surrogate of itaconate, 4-OI, during the challenge phase attenuates the manifestations of experimental HDM-induced asthma. **a** WT BALB/c mice were sensitized with HDM and received intranasal 4-OI (200 μg/mouse) or vehicle at the challenging phase before endpoint analysis on day 14. **b** Enumeration of total CD45^+^ cells, CD11b^+^ Siglec-F^+^ eosinophils and CD11b^+^ Ly-6G^+^ neutrophils in BAL and lung. **c** The bar chart show BAL levels of CCL24 in 4-OI or vehicle-treated mice. **d** Serum HDM-specific IgE and IgG1 levels and **e** Scatter plot with bar show cytokine secretion by ex vivo cultures of MedLN cells that had been re-stimulated with HDM (100 μg ml^−1^). **f** Representative images show histopathological sections of 4-OI- and vehicle-treated^*-*^ asthmatic lung stained with PAS. *Scale bars*, 100 μm for ×100 and 50 μm for ×400 images. **g** Enumeration of PAS^+^ airways from histopathological lung sections. Pooled data from two independent experiments are shown (*n* = 5–10 mice per group). *P < 0.05, 4-OI versus vehicle-treated control mice, unpaired *t-*test. *4-OI*, 4- octyl itaconate.

Lastly, to support the concept of utilizing the TCA cycle metabolite itaconate as a potential anti-asthmatic biologic, we assessed whether exogenous administration of 4-OI in vivo could ameliorate airway inflammatory response in WT mice (Fig. 8a). The cell-permeable itaconic acid derivative 4-OI is less thiol-reactive than other derivatives such as dimethyl itaconate (DI) and mediates no obvious toxicity issues^33^. As shown in Fig. 8b, HDM-sensitized mice that received 4-OI and HDM challenges intranasally following allergic sensitization demonstrated a reduced number of CD45^+^ leukocytes and eosinophils in BALF as well as in the lung. 4-OI treatment also significantly reduced CCL24 levels in BALF (Fig. 8c) and serum HDM-specific IgE (Fig. 8d). HDM restimulation of MedLN from 4-OI treated mice showed decreased IL4, IL5, and IL13 productions, whereas the IL17A level was not statistically significant (Fig. 8e). Lung histopathology and frequency of PAS^+^ airways showed decreased inflammatory cell infiltrate in peribronchial tissue and mucous cell metaplasia, respectively, in mice receiving 4-OI compared with vehicle-treated mice (Fig. 8f, g). Overall, these data demonstrate that exogenous supplementation of itaconate derivatives diminished the manifestation of HDM-induced type 2 airway inflammation in asthma.

## Discussion

DCs are bonafide antigen-presenting cells and play a pivotal role in developing T cell-mediated adaptive immune responses to inhaled aeroallergen HDM in allergic asthma. Efficient antigen recognition, activation, and migration of airway DC to draining MedLN are prerequisites for effective T cell priming and lung adaptive immunity^50,55,56^. Emerging evidence suggests that not only do metabolic pathways tightly regulate the immune-priming function of DCs, but their endogenous key metabolites can elicit a specific effector response^52,57,58^. Activated mature DC exhibit enhanced lysosomal acidification and undergoes rapid metabolic adaptations to expand endoplasmic reticulum (ER) and Golgi for effective antigen presentation and T cell priming^59–62^. Metabolic adaptations in activated DC, such as citrate withdrawal, support fatty acid biosynthesis to promote membrane expansion for efficient antigen presentation and induce pro- and anti-inflammatory mediator productions^59,63^. Recent work suggests an allergen-induced metabolic reprogramming in DC fine-tune the character of inflammatory responses, skewing it predominantly toward type2-high eosinophilic and type2-low Th17-mediated neutrophilic asthma^30,52,53,64^. We have recently demonstrated that induction of the glycolytic enzyme PKM2 is critical in the airways, particularly during acute asthma exacerbation^53^. Moreover, DMF, a surrogate of TCA cycle intermediates, interferes with DC migration and impairs HDM-induced airway type 2 inflammation^39^. However, the role of the mitochondrial *Irg1/*itaconate pathway corresponding to DC effector function in mediating allergic asthma remains unknown.

*Irg1* is one of the interferon-stimulated genes (*ISGs*) and is associated with type I IFN-mediated bacterial and viral killing by catalyzing the production of itaconic acid^33,36,38^. In addition, *Irg1* expression by myeloid cells mediates neutrophil-dependent immunopathological changes in *M. tuberculosis*-induced lung infection^28^. Furthermore, the cell-permeable derivative of itaconic acid (4-OI) exerts an immunomodulatory function by limiting pathogenic inflammation and oxidative stress due to its ability to modify the cysteine residues in target proteins, including the master antioxidant transcription factor Nrf2 and key metabolic regulator glyceraldehyde 3-phosphate dehydrogenase (GAPDH)^33,65^. Interestingly, *Irg1* influences MHCI expression and antigen presentation by inducing transporter expression associated with antigen processing 1 (TAP1) and proteasome subunit beta 9 (PSMB9)^66,67^.

This study investigated whether *Irg1*/itaconate pathway contributes to DC-mediated allergic sensitization and airway type 2 inflammation. Using two murine models of allergic asthma, we demonstrate that *Irg1* is induced in response to HDM sensitization and further by stimulation of STING pathway with second messenger c-di-GMP resulting in airway type 2-high eosinophilic inflammation and type 2-low mixed granulomatous inflammatory response, respectively. Interestingly, *Irg1* induction in antigen-presenting DC, not lung epithelial or myeloid cells, facilitates HDM-induced airway inflammation. Metabolic reprogramming of DC during maturation directly regulates MHCII-restricted antigen presentation, driving CD4+ T cell activation and producing Th2 cytokines^61,62^. Therefore, we hypothesized that manipulating the *Irg1*/itaconate pathway in DC might influence airway type 2 inflammation to inhaled HDM.

First, we showed that lack of the *Irg1/*itaconat*e* pathway drives an augmented eosinophilic airway inflammation and Th2 immune response to HDM. Restimulation of MedLN cells with HDM from *Irg1*^*−/−*^ mice showed increased IL4 and IL13 cytokine production that plays a critical role in type 2 eosinophilic airway inflammation and mucous cell metaplasia^68,69^. We showed that *Irg1*^*−/−*^ mice had an inadequate response to HDM sensitization, as manifested by elevated HDM-specific IgE and IgG1 serum levels compared to WT mice. Furthermore, *Irg1*^*−/−*^ DC in the MedLN from HDM-challenged mice had increased CD80 expressions (co-stimulatory molecule essential for T cell signaling via CD28 binding), CD205 (necessary for antigen recognition and uptake), Dectin2 (that sense HDM), and CCR7 (chemokine receptor that is required for migration of DC to MedLN with T cell priming)^70–73^. Second, a similar phenotype of accelerated eosinophilic airway inflammation and Th2 immune responses to HDM challenge was observed in recipient WT mice that received *Irg1*^*−/−*^ BMDC compared to recipient WT mice obtaining donor WT BMDC. Collectively, these results highlight the tight regulation of the *Irg1/*itaconate pathway in DC, driving the airway type 2 inflammation to HDM.

Emerging evidence suggests that pathological events that promote Th2 signaling pathways and inflammation in asthma have been associated with oxidative stress and mitochondrial redox metabolism^74–76^. Superoxide anion reactive oxygen species (ROS) are generated as a byproduct of mitochondrial oxidative phosphorylation in response to allergen stimulation and STING pathway activation^77,78^. Consistent with this, we demonstrate that lack of *Irg1*/itaconate axis in DC leads to overall impaired redox status as evidenced by reduced expression of SOD1 and Nrf2, increased oxidative metabolism, and greater mitochondrial superoxide production. In a potential mechanistic link, we demonstrate that enhanced antigen presentation capabilities of OVA-peptide-pulsed BMDC isolated from *Irg1*^*−/−*^ mice compared to WT control mice, as manifested by increased proliferation of naïve OT-II cells and Th2 effector cytokine (IL4, IL5, and IL13) productions in coculture experiments. Thus, increased mitochondrial redox and oxidative stress might contribute in part to the phenotype of augmented antigen presentation capabilities of *Irg1*^*−/−*^ DC.

Itaconate serves as electron-pair acceptors and is susceptible to nucleophilic attack by nucleophiles such as glutathione (GSH). The cell-permeable octyl ester derivative 4-OI is metabolized intracellularly to itaconate and exerts potent anti-inflammatory and antioxidant effects through several mechanisms, which includes mitochondrial SDH inhibition via reducing intracellular ROS generation, alkylation of redox-sensing protein KEAP1 that releases Nrf2 to induce transcription of the antioxidant genes, blocking aerobic glycolysis by inhibiting the enzyme activity of GAPDH and activation of transcription factor ATF3^33,34,65,79^. Although quantification of polar metabolites in HDM-pulsed and stimulation with STING agonist c-di-GMP accumulated itaconate in DC, we found that the endogenous production of itaconate was not sufficient to protect the mitochondrial oxidative damage. However, our results showing that exogenous 4-OI treatment significantly reduced OCR and mitochondrial superoxide generation in DC via Nrf2-dependent mechanism agrees with previous studies^33,54,80,81^, suggesting that exogenous surrogate of itaconate application in part replenish the endogenously-produced itaconate thereby prevents mitochondrial oxidative damage.

We also assessed whether exogenous administration of itaconate surrogates might represent an effective therapeutic approach to asthma. Prior studies have found that 4-OI administration provides significant protection from lethal endotoxemia in mice and reduces proinflammatory cytokine release^33,82,83^. Perhaps most tellingly, we show that 4-OI treatment following HDM sensitization once T cell priming had already occurred attenuated airway inflammatory response, mucous cell metaplasia, and Th2 effector cytokine productions. Thus, we propose developing novel therapeutic candidates targeting the *Irg1*/itaconate pathway for allergic airway type 2 inflammation. Our current study suggests a critical role for the *Irg1*/itaconate pathway in modulating DC effector function regulating Th2 immune responses to HDM in an experimental murine model of allergic asthma. Elucidating the *Irg1*/itaconate pathway in non-immune cells and structural cells from different lung compartments is further warranted.

**Fig. 9:**
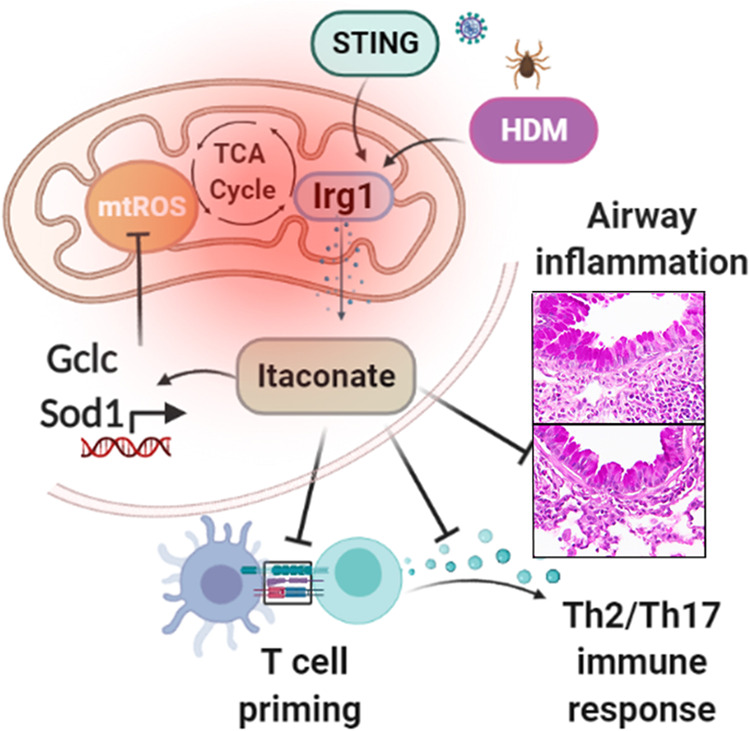
Proposed mechanisms of *Irg1*/itaconate pathway in airway inflammation. Allergen (HDM) or upper respiratory viral infections (STING pathway activation) progressively induce *Irg1* expressions in DC via a mitochondrial superoxide-dependent pathway, impairs effective antigen presentation and airway inflammatory response. The TCA cycle metabolite itaconate attenuates mitochondrial ROS production via inductions of antioxidative programs, which in turn influences DC effector functions and limit the development of airway inflammation and T cell-mediated allergic immune responses in asthma.

Overall, our results identify a novel function for the *Irg1*/itaconate pathway in DC that is essential for antigen priming and effector function, subsequent induction of allergic sensitization, and type 2 airway inflammation in the lung (Fig. 9). We demonstrate that HDM alone or in combination with innate STING pathway stimulation induces the *Irg1* expression and itaconate production via mitochondrial superoxide generation. Cysteine-modifying itaconate derivative 4-OI promotes an anti-inflammatory mechanism by reducing mitochondrial oxidative damage in DC. Furthermore, we demonstrate that lung administration of 4-OI attenuates the hallmarks of allergic asthma manifestation, which includes HDM sensitization, Th2-mediated eosinophilic airway inflammation, and mucous cell metaplasia. Therefore, these findings suggest the *Irg1*/itaconate pathway in the lung as a novel metabolism-based therapeutic target for patients with allergic asthma, especially those with type2- high airway inflammation.

## Methods

### Mice

*Irg1*^*−/−*^^9,17,84^, wild type control (C57BL/6NJ), BALB/c, and B6.Cg-Tg(Tcra Tcrb)425Cbn/J (transgenic mice expressing MHCII-restricted TCR that binds to OVA _323-339_ peptide antigen^85^, all available from The Jackson Laboratories (Bar Harbor, ME). The mutant strain carrying the Acod1^em1 (IMPC) J^ allele (*Irg1*^*−/−*^) are viable and fertile. Six- to eight-week-old male and female mice were utilized for studies. Experimental protocols of the murine model of allergy and asthma were approved by the Animal Care and Use Committee of the Auburn University, Auburn, AL.

### HDM sensitization and challenge models

WT (BL6N/J) and *Irg1*^*−/−*^ mice were injected with two intraperitoneal injections of low-endotoxin HDM (100 μg) (*Dermatophagoides pteronyssinus* extract, Greer Laboratories, Lenoir, NC) or sham-PBS with aluminum hydroxide (40 mg ml^−1^, Invivogen, San Diego, CA) on day one and day four and challenged with HDM (50 μg per mouse, i.n) or sham-PBS on days 8, 10, 12 before harvest on day 14^86^. In separate experiments, BL6/J mice were sensitized with HDM alone (50 μg dissolved in 40 µl volume of sterile PBS; *i.n*) and in combination with STING pathway agonist (c-di-GMP; Biolog Life Science, Bremen, Germany; 5 μg per mouse) on days 1, 3, and 5. Mice were challenged in two phases with four-day intervals. Intranasally, HDM was administered thrice on alternate days (days 11 through 13 and 17 through 19). Additionally, mice receive STING agonist ci-di-GMP (5 μg per mouse) twice at the beginning of each challenge phase on days 11 and 17 before harvest on day 20. In separate control groups, mice receive PBS or STING agonist ci-di-GMP only. For in vivo 4 octyl itaconate (4-OI)-study, 6-weeks old BALB/c mice of both sex were sensitized with low-endotoxin HDM and alum (*i.p*.) on days 0 and 4 and HDM-challenged (25 µg per mouse in a volume of 40 μl sterile PBS) on days 8, 10 and 12 before harvest on day 14. Mice were treated with 4-OI (10 mg kg^−1^ body weight) (Tocris, Minneapolis, MN) or vehicle (2.0% DMSO in PBS) 5 h before HDM challenge during each allergen challenge on days 8, 10, and 12.

### Adoptive transfer of HDM-pulsed BMDC to naïve mice

Bone marrow cells from WT (BL6N/J) and *Irg1*^*−/−*^ mice were cultured at 1 × 10^6^ cells ml^−1^ in Iscove modified Dulbecco medium (Gibco/Life Technologies, CA) containing 10% heat-inactivated FCS, penicillin-streptomycin (100 U ml^−1^), L-glutamine (2 mM), 2-mercaptoethanol (50 µM), recombinant mouse granulocytes macrophage colony-stimulating factor (20 ng ml^−1^) and recombinant mouse IL-4 (10 ng ml^−1^, Peprotech) as described below, and were used for adoptive transfer experiments. On day 7, floating non-adherent bone marrow-derived dendritic cells (BMDC) were HDM- (100 µg ml^−1^) or sham-pulsed with PBS for 16 h at 37 °C. Live 0.2 × 10^6^ cells were adoptively transferred in 20 µl of PBS through intranasal administration on day 0 to recipient naïve WT (BL6N/J) mice. WT recipient mice received daily HDM challenges (50 µg, i.n) on days 9, 11, 13, 15, and were sacrificed on day16 as previously^86,87^. Three independent experiments were performed for BMDC adoptive transfer model.

### BAL and lung histopathology

Bronchoalveolar lavage (BAL) was collected with 0.5 ml PBS and red blood cells were lysed with ACK buffer for 2 min at 4 °C, as previously described^49^. BAL cells were re-suspended in 0.3 ml RPMI-1640 containing 10% FBS, and cell counts were performed using a hemocytometer, while differential cell counts were performed by flow cytometry. Lungs were fixed in 10% formalin for 24 h, dehydrated through gradient ethanol, and embedded in paraffin. Sections were cut sagittally at a thickness of 5 µm and stained with hematoxylin and eosin and periodic acid Schiff (PAS).

### Restimulation of ex vivo cultures of MedLN cells

Single-cell suspensions from mediastinal lymph nodes (MedLN) of mice were prepared using sterile RPMI medium containing 10% FBS. MedLN cells were counted and cultured at a density of 0.3 × 10^6^ cells ml^−1^ in round-bottom 96-well plates and pulsed with HDM (100 µg ml^−1^) for 96 h at 37 °C in RPMI medium containing 10% FBS. In selected experiments, cells were treated with 4-OI (50 µM). The concentrations of IL4, IL5, IL13, IFN-γ, and IL17A in the supernatants were measured using sandwich ELISA kits (ThermoFisher Scientific) with sensitivity limits of 4 pg ml^−1^ for IL-4, IL-5, IL13, IL17A.

### HDM-specific IgE and IgG1

HDM-specific serum IgE and IgG1 levels were determined, as previously described^48^. Overnight coated 96-well plates (0.01% HDM in PBS) were blocked with 1% BSA in PBS. Serum samples were added to the plates for one hour, washed 6X with PBS with 0.05% Tween‐20 before incubation with biotinylated anti-mouse IgE or IgG1 (Pharmingen, San Jose, CA) for 1 h. Plates were washed six additional times, and streptavidin-HRP (R&D Systems, Minneapolis, MN) was added for 30 min. The addition of TMB substrate determined bound HDM-specific antibodies.

### Flow cytometry

Lungs cells were isolated by enzymatic digestion using type IV collagenase, 1 mg ml^−1,^ and DNase I (Worthington, Lakewood, NJ) 0.1 mg ml^−1^ at 37 °C for 30 min with agitation. Single-cell suspension from digested lungs was incubated with Fc Block^TM^ (BD Pharmingen, San Jose, CA) to react with Fcγ III/II receptors before surface staining in staining buffer (containing PBS, 3% FBS, 2 mM EDTA, and 10 mM 4‐(2‐hydroxyethyl) ‐1‐piperazineethanesulfonic acid) at 4 °C for 10 min. Cell samples were first stained with Live/dead Fixable Violet Staining Kit (Invitrogen) in 50 μL PBS at 4 °C in the dark for 15 min. Eosinophils (CD45^+^ CD11b^+^ SiglecF^+^ Ly6G^−^) and neutrophils (CD45^+^ CD11b^+^ SiglecF^−^ Ly6G^+^) in BAL and lung were identified using the following antibodies: rat anti-mouse CD45 efluor 450 (clone 30-F11), CD11b-APCCy7 (clone M1/70) Ly6G-APC (clone 1A8) and SiglecF-PE-Texas Red (clone 1RNM44N). DC-specific activation markers were identified with rat anti-mouse CD11c-APC-eFluor780 (clone N418), I-A/I-E (MHC-II)-eFluor450 (clone M5/114.15.2), CD11b-PE-TexasRed (M1/70.15), Siglec F-eFluor660 (1RNM44N), CD86-PE (clone, PO3), CD80-PE-Cy5 (clone, 1610A1), CCR7- APC (clone, 4B12), Dectin 1-PE (clone CLEC7A), Dectin2-AF647 (cloneD2.11E4), TLR4-APC (clone SA15-21), and CD205-PerCP/Cy5.5 (clone NLDC-145). For analysis of intracellular cytokines, cells were first stained with surface antigens against rat anti-mouse CD45 efluor 450 (clone 30-F11), CD3-Alexa Fluor 647 (clone 17-A2), and CD4-APC-eFluor 780 (clone GK1.5) and fixed with IC Fixation Buffer (eBiosciences) for 30 min, followed by a wash with Perm/Wash buffer (BD). Cells were then re-suspended and reacted to monoclonal antibody cocktails of rat anti-mouse IL-4 PE-Cyanine7 (11B11), IL-5 PE (TRFK5), IL-13 Alexa Fluor 488 (eBio13A), IL-17 eFluor 450 (eBio17B7), IFN-γ-PerCP-Cyanine5.5 (clone, XMG1.2), and in Perm/Wash buffer (50 μl) for 30 min at room temperature, and viable CD3^+^/ CD4^+^ cytokine^+^ cells were quantified using FMO (fluorescence minus one) as controls. MedLN cells were first stained with surface markers for CD3-Alexa Fluor 647 (clone 17-A2), CD4-FITC (clone GK1.5), and CD25-PE-Cy7 (clone PC61.5) and fixed and permeabilized using Foxp3 staining buffer (eBiosciences) and reacted to Foxp3-PE antibody (clone NRRF-30) to quantify Tregs. To analyze the Irg1 expression on lung DC, cells were first stained with surface antigens (CD11c, CD11b, MHCII, and SiglecF) and perm/fixed for intracellular staining. Cells were then re-suspended and reacted to monoclonal *Irg1* (clone: EPR22066, Abcam, Cambridge, MA) or IgG isotype control (clone: EPR25A). antibodies and probed with AF680 fluorophore-labeled secondary antibody (A32734, ThermoFisher). Data were acquired on an LSRII (BD Biosciences, USA) equipped with 407, 488, and 633 LASER lines using DIVA 8 software and analyzed with the Flow Jo software version 10.7.1 (Treestar, San Carlos, CA). Using FSC/SSC plot, cellular debris was excluded.

### BMDC culture

Femur bones were isolated from euthanized WT mice and cultured at a density of 1 × 10^6^ cells ml^−1^ in Iscove modified Dulbecco medium (Gibco/Life Technologies, CA) containing 10% heat-inactivated FCS, penicillin-streptomycin (100 U ml^−1^), L-glutamine (2 mM), 2-mercaptoethanol (50 µM), recombinant mouse granulocytes macrophage colony-stimulating factor (20 ng ml^−1^) and recombinant mouse IL-4 (10 ng ml^−1^, Peprotech). Equal volumes of medium were supplemented (day 3 and day 5), and non-adherent cells were collected on day 6 and plated in 6-well tissue culture plates (2 × 10^6^ cells/well) in 2 ml of differentiation media. Cells were stimulated with HDM (100 µg ml^−1^) alone and with c-di-GMP (10 µM) in the presence of 5% FBS containing media without GMCSF and IL-4 for 16 h.

### Polar metabolomics profiling

BMDCs were harvested post 16 h of HDM (100 µg ml^−1^) alone and/or with c-di-GMP (10 µM) stimulation and processed for polar metabolite profiling as described earlier^88^. Briefly, 4 ml of 80% methanol (vol/vol) was added. The plate was incubated at −80 °C for 15–20 min, and the cell lysate/methanol mixture was collected. Metabolite containing supernatant was collected by centrifugation @ 14,000 *×* *g* for 5 min at 4 °C. Further, 500 μl of 80% methanol (−80 °C) were added to 15 ml tubes and repeated the extractions. The extractions samples were pooled, dried (speed vac), and stored at −20 °C until analysis. Extracted metabolites were run using a 5500 QTRAP LC-MS/MS system via SRM followed by Q3 peak area integration using MultiQuant 2.0 software (Mass Spectrometry Core, Beth Israel Deaconess Medical Center). This peak area output was considered raw data from metabolomics profiling and processed with MetaboAnalyst 5.0 software and IPA analysis (Ingenuity Pathway, Qiagen).

### Mitochondrial respiration assay

Oxygen consumption rate (OCR) of BMDC isolated from WT and *Irg1*^*−/−*^ were measured using an XFp extracellular flux analyzer (Seahorse Bioscience). BMDC were seeded at a density of 120,000 cells per well in XFp mini-culture plates in IMDM medium containing 5% FBS at 37 °C and pulsed with HDM (100 µg ml^−1^) overnight before the assay. In separate experiments, cells were pulsed with HDM and c-di-GMP (5 µM) and cultured overnight in the presence and absence of different 4-OI concentrations (50–100 µM). Cells were then incubated in an assay medium (DMEM with 25 mM glucose, 1 mM pyruvate and 2 mM sodium bicarbonate, pH~ 7.4) for at least 30 min in the non-CO_2_ incubator before the assay. Sequential injections of oligomycin (1 mM, mitochondrial ATP synthase inhibitor), carbonyl cyanide 4-(trifluoromethoxy) phenylhydrazone (FCCP, 2 mM, uncoupler of OXPHOS from ATP synthesis), and antimycin A/rotenone (0.5 mM, ETC inhibitor) were delivered after basal measurements from ports of the system. The effects on OCR were recorded 3 times every 5 min interval, and data were analyzed using Wave software 2.6.1 (Seahorse Bioscience) after normalization with total protein.

### Measurement of mitochondrial superoxide

BMDC from WT and *Irg1*^*−/−*^ mice were incubated with 5 µM MitoSOX (Molecular Probes, USA) in DPBS (1X) for 30 min at 37 °C. Oxidation of mitochondrial superoxide with MitoSOX Red produces 2-hydroxyethidium (a derivative of Mitosox Red indicator) detected by increased fluorescence in the FL2 emission channel (585/42 nm). Levels of mitochondrial superoxide generated after HDM (100 µg ml^−1^) and c-di-GMP (5 µM) pulse and in response to treatment with 4-OI (50 µM) were quantified. The data were analyzed by subtracting the background MFI of the nonfluorescent sample (negative control) from the measured MFI values of fluorescent samples in the flow cytometry.

### Antigen uptake and migration assay

WT and *Irg1*^*−/−*^ mice received intranasal administration of Alexa fluorophore 647-labeled OVA (100 µg per sensitization) dissolved in 40 µl of PBS. At 72 h after sensitization, migrating DC were enumerated in digested lungs and MedLN as SiglecF^−^ CD11c^+^ MHCII^hi^ CD11b^+^ AF647-OVA^+^ cells by flow cytometry.

### OT-II coculture

Ex vivo antigen-specific T-cell proliferation was assessed using CFSE-labeled splenic CD4^+^ T cells obtained from naïve (B6.Cg-Tg(Tcra Tcrb)425Cbn/J) transgenic mice (Jackson Laboratories, Bar Harbor, ME) in coculture to determine the antigen presentation capability of DC. The mice express a transgenic MHCII-restricted TCR and recognize the OVA peptide antigen^85^. Splenic naive CD4^+^ T cells were purified using EasySep Mouse CD4^+^ T Cell Isolation Kit (Stem cells, Vancouver, CA) and were labeled with 5 µM CFDA-SE (carboxyfluorescein diacetate succinimidyl ester; Cayman Chemical, MI, USA) in DPBS for 20 min at 37 °C. BMDC from WT and *Irg1*^*−/−*^ mice were pulsed overnight with 5 μg ml^−1^ of OVA_323-339_ peptide (AnaSpec, Fremont, CA, USA) or PBS. 1 × 10^5^ OVA peptide-specific CD4^+^ OT-II cells were cocultured with 2 × 10^4^ CD11c^+^ BMDCs for 4 days in 96-well round-bottom plates. T cell proliferation was quantified by flow cytometry using the CFSE dye dilution method. The FlowJo Proliferation Platform analyzed gated CD4+ T-cells as percent divided. The quantity of IL4 and IL13 released into the culture medium was measured using ELISA. Additionally, cells were stained with CD3-Alexa Fluor 647 (clone 17-A2), CD4-APC-eFluor 780 (clone GK1.5) before incubation with GATA3 eFluor 450 (clone TWAJ) for flow cytometry.

### Calriom S mouse microarray analysis

Total lung RNA was isolated using PureLink R.N.A. Mini Kit (Invitrogen) according to the manufacture’s instruction and probed using the Clariom S Mouse Array (Applied Biosystems) at the facility (AKESOgen Inc, GA, USA). Raw data that included feature intensity values were converted into summarized expression values using Robust Multi-array Average (RMA), consisting of background correction, quantile normalization, and summarization across all samples. All samples passed QC thresholds for hybridization, labeling, and the expression of housekeeping gene controls (Ake). Principal component analysis was performed for detecting outliers across all chips. Gene expression data generated from Clariom S Mouse arrays were analyzed. Differentially expressed genes (DEGs) were identified using R Project for Statistical Computing, Partek Flow, and Transcriptome Analysis Console (Applied Biosystems) packages. A false discovery rate (FDR), *P* value of less than 0.05, was considered significant. The data generated in this publication have been deposited in the NCBI Gene Expression Omnibus and are accessible through GEO Series accession number GSE165969.

### RT-PCR

RNA was isolated from lung cells (magnetically sorted using mouse pan-DC or mouse epithelial cell enrichment kit; Stem cells, Vancouver, CA) or BMDCs using trizol reagent (Life Technologies, Grand Island, NY, USA), and cDNA was synthesized by High Capacity RNA-to-cDNA kit (Applied Biosystems). qPCR for *Irg1* (Applied Biosystems probe Mm01224532_m1) and β-actin (Applied Biosystems probe Mm02619580_g1) were performed using Taqman gene expression assay (Life Technologies). Gene expression was quantified by SYBR green using following primers: *SOD1*, Forward: 5’-CACTCTAAGAAACATGGTGG-3’ and Reverse: 5’-GATCACACGATCTTCAATGG-3’; *Nox1*, Forward: 5’-CAGAAGACCCTTAAGAAAACC-3’ and Reverse: 5’-GTGCTGAACTCCTCAATATG-3’; *Nrf2*: Forward: 5’-CATTCCCGAATTACAGTGTC-3’ and Reverse: 5’-GGAGATCGATGAGTAAAAATGG-3’; *Gclm*: Forward: 5’-GCACAGGTAAAAACCCAATAG-3’ and Reverse: 5’-TTAGCAAAGGCAGTCAAATC-3’; *Gclc*: Forward: 5’-CTATCTGCCCAATTGTTATGG-3’ and Reverse: 5’-ACAGGTAGCTATCTATTGAGTC-3’; *Hif-1α*: Forward: 5’-GGTTCCAGCAGACCCAGTTA-3’ and Reverse: 5’-AGGCTCCTTGGATGAGCTTT-3’; *Glut-1*: Forward: 5’-CATCCTTATTGCCCAGGTGTTT-3’ and Reverse: 5’-GAAGACGACACTGAGCAGCAGA-3’; *Ldha*: Forward: 5’-CACTGACTCCTGAGGAAGAGGCCC-3’ and Reverse: 5’-AGCTCAGACGAGAAGGGTGTGGTC-3’, *36B4*: Forward: 5’-GGACCCGAGAAGACCTCCTT-3’ and Reverse: 5’-GCACATCACTCAGAATTTCAATGG-3’. After amplification, C_t_ values were analyzed (2^−ΔΔCt^ method) and expressed relative to the housekeeping genes.

### Immunoblot

Total cell lysates were prepared from BMDC cultures using Pierce IP lysis buffer (25 mM Tris-HCl pH 7.4, 150 mM NaCl, 1% NP-40, 1 mM EDTA, 5% glycerol with complete protease inhibitors) and concentration was determined (Pierce B.C.A. kit, ThermoFisher Scientific). Forty micrograms of protein that had been separated by SDS-PAGE using 4–12% Bis-Tris gels (Lonza, Walkersville, MD) was transferred to a nitrocellulose membrane. Membranes were reacted with anti-rabbit Gclc (1:500 dilution) (ThermoFisher Scientific), Irg1, and Sod1 monoclonal antibodies (1: 1000 dilution) (Cell signaling Technology, Danvers, MA) and probed with anti-rabbit β-actin before developing in Odyssey system (Li-COR).

### Statistics

Data were analyzed using Graph Pad Prism version 7.0b using an unpaired *t*-test for normally distributed data and a Mann–Whitney test for non-normally distributed data. We analyzed multiple comparisons using a one-way ANOVA with Sidak’s multiple comparison test or a two-way ANOVA. Data are presented as mean ± SD. A *P* value <0.05 was considered significant.

## Supplementary Information

Supplementary Figures
