# Supplementary material for: Irg1/itaconate metabolic pathway is a crucial determinant of dendritic cells immune-priming function and contributes to resolute allergen-induced airway inflammation
Source: Mucosal Immunol. 2021 Oct 20;15(2):301–13. doi: 10.1038/s41385-021-00462-y (PMC8866123; doi:10.1038/s41385-021-00462-y)
Supplement: Supplementary file 1 — Supplementary Figures [file 41385_2021_462_MOESM1_ESM.pdf]

Supplementary Figure 1.

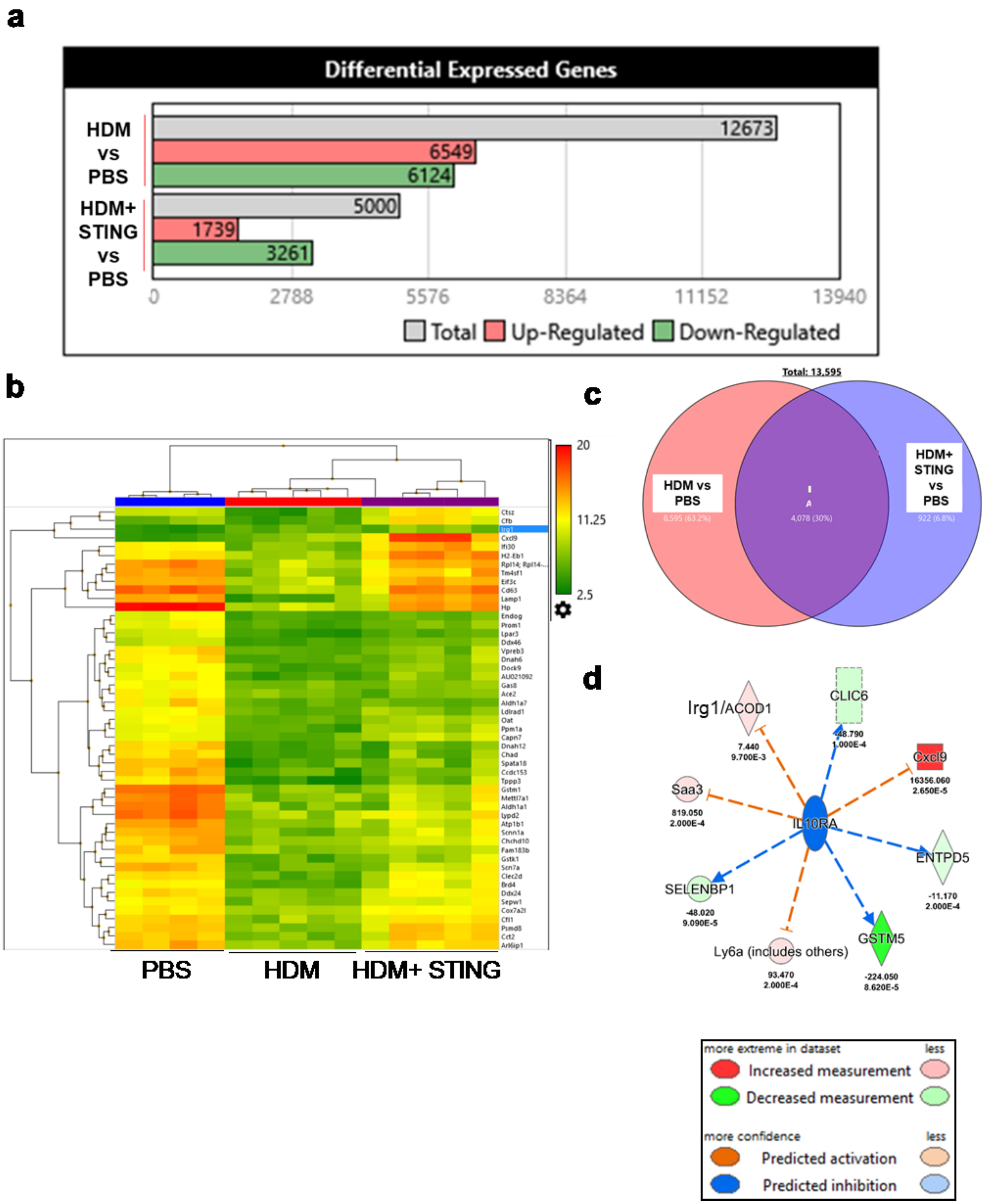

Microarray analysis of asthmatic lungs. **(a)** Summary of Differentially Expressed genes (DE, |Fold Change| >2; p-value < 0.05) derived from Gene expression profiling (GEP) data analysis; (**b)** Heatmap with the top 50 most DE genes based on three groups ANOVA. (**c)** The Venn diagram shows a comparison representing DE genes in Control PBS vs. HDM and HDM + STING-induced asthmatic lungs, respectively. **(d)** Top upstream network predicted by IPA based on most significant DE genes in HDM+ STING vs. PBS group.

Supplementary Figure 2.

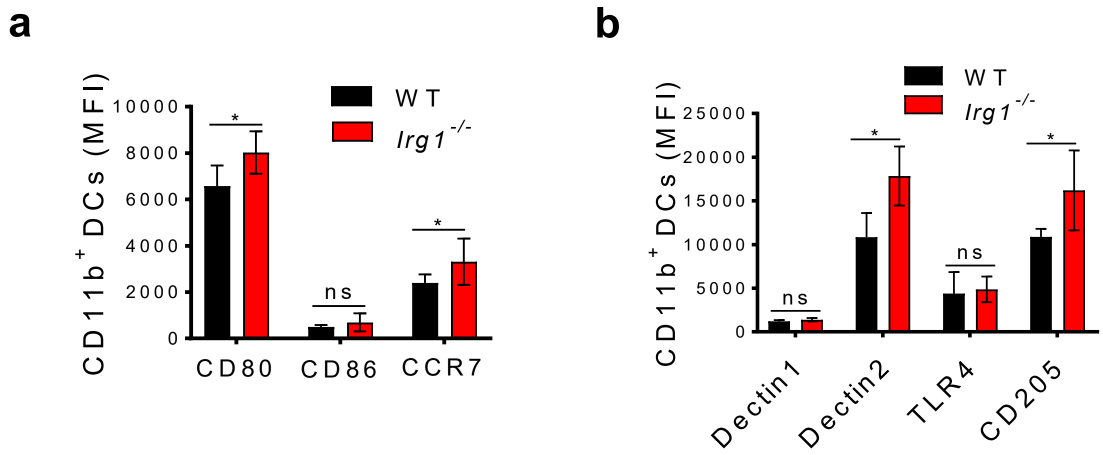

Characterization of HDM-challenged *Irg1*$^{-/-}$ migratory CD11b^{+}DCs in draining MedLN. Mean fluorescence intensity of CD11b^{+} DC from HDM-challenged WT and *Irg1*$^{-/-}$ mice **(a)** Activation markers (CD80, CD86, and CCR7) and **(b)** CLRs (Dectin 1 and 2), TLR4 and CD205. Data represent the mean +/- S.D. one of two independent experiments. (n= 4-6 mice) *$P < 0.05$ WT versus *Irg1*$^{-/-}$, unpaired *t*-test.
